# The impact of a hospital electronic prescribing and medication administration system on medication administration safety: an observational study

**DOI:** 10.1186/s12913-017-2462-2

**Published:** 2017-08-09

**Authors:** Seetal Jheeta, Bryony Dean Franklin

**Affiliations:** 1 Centre for Medication Safety and Service Quality, Imperial College Healthcare NHS Trust, London, UK; 2 Research Department of Practice and Policy, UCL School of Pharmacy, London, UK

**Keywords:** Electronic prescribing, Observational study, Medication errors, Hospital

## Abstract

**Background:**

The aim of the study was to explore the impact of the implementation of an electronic prescribing and medication administration system (ePA) on the safety of medication administration in an inpatient hospital setting. Objectives were to compare the prevalence and types of: 1) medication administration errors, and 2) documentation discrepancies, between a paper and an ePA system. Additionally, we wanted to describe any observed changes to medication administration practices.

**Methods:**

The study was based on an elderly medicine ward in an English hospital. From December 2014 to June 2015, nurses’ medication administration rounds were observed every 5 days before and after ePA implementation using an interrupted time-series approach. Medication administration error and documentation discrepancy rates pre- versus post-ePA were analysed descriptively and chi-squared tests used to test for any difference; segmented regression analysis was used to determine changes in longitudinal trend.

**Results:**

Observations were made at 15 pre- and 15 post-ePA implementation time-points. Pre-ePA on paper, there were 18 medication administration errors in 428 opportunities for error (4.2%; 95% confidence interval 2.3–6.1%), and with ePA there were 18 in 528 (3.4%; 95% confidence interval 1.9–5.0%; *p* = 0.64). Regarding documentation, pre-ePA on paper there were 5 discrepancies in 460 observed documentations (1.1%; 95% confidence interval 0.1–2.0%); with ePA there were 18 in 557 (3.2%; 95% confidence interval 1.8–4.7%; *p* = 0.04). The most common electronic documentation discrepancy was documentation that a dose had been administered when it had not. Segmented regression analysis was unable to detect any significant longitudinal changes. Changes to working practices post-ePA were observed, such as nurses demonstrating less-consistent self-checking when preparing and administering medications.

**Conclusions:**

Findings suggest no change in medication error rate, although ePA encourages certain types of errors and mitigates others. There was a statistically significant increase in documentation discrepancies which is likely to be due to adoption of new working practices with ePA.

**Electronic supplementary material:**

The online version of this article (doi:10.1186/s12913-017-2462-2) contains supplementary material, which is available to authorized users.

## Background

The use of electronic prescribing and administration (ePA) systems has been shown to significantly improve patient safety by reducing medication errors [1–4]. Although ePA has been established in the United States (US) for some years, its uptake in the United Kingdom (UK) has been slower; in 2011 only 13% of English hospitals had ePA across most inpatient areas [5]. Nevertheless, the UK government is committed to advocating its use more widely, which is expected to increase considerably [6, 7].

In the UK, medication administration errors (MAEs) occur in an estimated 5.6% of non-intravenous doses and 35% of intravenous (IV) doses [8]. Most studies exploring the impact of introducing ePA have focussed on prescribing rather than medication administration [1]. Other studies report the impact of bar-code medication administration technology [9, 10], but the findings are not applicable to ePA systems where bar-coding is not utilised. Therefore, the impact of ePA on medication administration safety is unclear, both in terms of positive impact on safety and potential negative unintended consequences [11–13]. Existing UK ePA studies focussing specifically on medication administration are limited and report mixed outcomes. One early study observed no difference in MAE rates with the use of electronic prescribing [14] while in contrast, Fowlie et al. [15] and Franklin et al. [16] reported reductions in MAEs, with the benefits in the latter study at least partly attributable to closed-loop automated dispensing integrated with ePA. Previous studies have used before-and-after designs and have not explored the longitudinal effect of ePA on MAEs following its implementation. A longitudinal time series approach would address some of the limitations associated with before-and-after studies, and allow exploration of the time period required for administration practices to normalise following ePA implementation.

Our aim was to explore the impact of ePA on the safety of medication administration in an inpatient hospital setting. Objectives were to measure the prevalence and types of: 1) MAEs, and 2) medication administration documentation discrepancies (DD), pre- and post-ePA. Additionally, we wanted to describe any observed changes in the medication administration process and explore any ‘settling in time’ for processes to normalise post-ePA.

## Methods

### Setting

We studied an elderly medicine inpatient ward in a large London teaching hospital. The ward was one of two wards implementing ePA prior to organisational rollout.

The ward comprised fourteen beds. There were generally three nurses on the ward. There were four main medication administration rounds each day, at 8 am, 1 pm, 6 pm and 10 pm. Pre-ePA, prescribers were required to hand-write details of the drug, dose, route, date and administration times on a pre-formatted paper drug-chart for each patient. Nurses used this for instruction of medication administration and to document details of doses given (or reason for omission). Paper drug-charts were generally located at each patient’s bedside. Individual bedside lockers stored patients’ own medication brought from home or dispensed by the hospital for that patient. Ward stock medication was stored in a lockable metal storage box that could be transported between patients.

Following implementation of a commercially available ePA system, prescribers entered medication details electronically. Nurses viewed the prescribed medications and documented details of dose administration or omission electronically; the system did not incorporate bar-code medication administration technology. Access to ePA required a secure log-in and password. During medication administration rounds, nurses wheeled a portable computer on wheels (COW) between patients. COWs were either standalone, or with integrated medication storage drawers. Post-ePA, other wards continued to use paper drug-charts. Therefore, patients often arrived on the study ward with a paper chart already in use and medication administration was conducted using paper until medication orders were transferred to ePA; a small proportion of post-ePA observations were therefore on paper.

### Study design

An interrupted time-series approach was used to explore the longitudinal effect of ePA on MAEs [17]. We planned to collect data at successive time-points every five days, including weekdays and weekends, for twelve time-points pre-ePA and at least twelve post-ePA [17]. This number of time-points is the recommended sample size for interrupted time-series studies of this nature [17]; it would also allow time for adjustment to new working practices and identification of any settling-in time. Ethics approval was not required; the study was registered locally as a service evaluation. The study has been reported according to recommended best practice for MAE studies [8].

### Data collection

At each time-point, one research pharmacist observed the busiest medication administration round of the day, which was at 8 am. Generally one nurse was observed at each time-point, although if a second nurse was still administering medication to their patients after the first had finished, their remaining administrations were also observed. The researcher observed the preparation and administration of medication and compared these with the prescribed medication to identify MAEs and DDs. Wherever possible, the researcher viewed medication orders before the dose was administered; when this was not possible, details of medication administered were recorded and checked against the order afterwards. The researcher tactfully intervened to prevent MAEs likely to result in harm from reaching the patient. Only regularly prescribed medications were assessed for omissions as the researcher was not able to judge the appropriateness of not administering medication orders prescribed to be given when required; once only doses were generally given outside of scheduled drug rounds. Intravenous medications were excluded as very few were administered on the study ward; inclusion of different numbers of intravenous doses pre- and post-EPA may have confounded the results as these are associated with higher MAE rates [8]. Administration of oxygen and dietary supplements was also excluded.

Data collection forms were developed and piloted beforehand. Field notes were made of any factors that may have influenced medication administration. Potential MAEs and DDs were documented and later verified through discussion with a second researcher.

### Data analysis

The MAE rate was calculated as the percentage of opportunities for error (OE) with one or more MAE; an OE could theoretically contain more than one MAE. To calculate the DD rate, the denominator was the number of observed documentations of dose administrations or omissions; for each medication order, there could be a maximum of one DD. Definitions of MAE, DD and OE are given in Table 1. Ninety-five percent confidence intervals (CI) were presented for MAE and DD rates. MAE and DD rates at each time-point were presented graphically and segmented-regression analysis used to identify any changes in level and trend post-ePA; chi-squared tests with Yates’ correction were also used to test for any differences pre- and post-ePA. Clinical severity of MAEs was not assessed.

**Table 1:** Definitions

Opportunity for error
The number of opportunities for error (OE) was the denominator used to determine the MAE rate. An OE was defined as any dose that was prepared and administered to the patient and could be determined as being correct or incorrect by the researcher, or a dose that was due for administration but omitted in error [16]. An administered drug was defined as either the patient consuming the prepared medication or the prepared medication being left by the patient’s bedside for self-administration [16]. Medication doses that were administered unsuccessfully due to the patient being unable to take the dose or subsequent refusal were also considered OEs if the researcher could determine preparation and attempted administration as being correct or incorrect. Each dose was considered to be one OE.
Medication administration error
Medication administration errors (MAE) were defined as any deviation or omission from the medication order as stated on the patient’s drug chart [16]. An omission was defined as a dose of medication that had not been administered by the time of the next scheduled dose. This included omission of a dose because the medication was not available on the ward as well as unintentional omissions. Doses omitted according to a doctor’s instructions, or if the patient was not on the ward or refused the medication, were not considered to be OEs. Doses omitted as a result of a nurse’s clinical judgement were also not considered OEs. Administration processes that did not adhere to local hospital policy were not considered MAEs; for example a dose not administered within two hours of the time for which it was prescribed was not considered an MAE if it was otherwise correct. Errors prevented by the observer were included as MAEs although errors prevented by other healthcare professionals working within their usual roles were not.
Documentation discrepancies
Discrepancies in documentation (DD) occurred when the documented details of dose administration or omission were different to what happened in practice as observed by the researcher [16]. Documentation was considered for all medication doses due, including omissions.

## Results

### Observations and opportunities for error

Data were collected from December 2014 to June 2015 at 15 time-points pre- and 15 post-ePA. After the first three time-points, data collection ceased for 4 weeks before resuming; this was due to temporary uncertainty around ePA implementation on the ward. Time-series analysis therefore only includes data collected after the temporary break (12 time-points pre- and 15 time-points post-ePA). In practice, data were collected every 3 to 6 days with a mean interval of 5 days. One data collection point during each of the pre- and post-ePA periods was missed due to the researcher’s unavailability.

There were 86 patient encounters observed pre-ePA and 86 post-ePA, where each patient had one encounter with a nurse at each time-point. Pre-ePA, 53 (11%) of 481 doses during observations were not considered OEs, there were therefore 428 OEs. Post-ePA, 46 (7%) of 643 doses were excluded as OEs, leaving 597 OEs. Of these, 528 (88%) were with ePA and 69 (12%) with paper. Additional file 1 gives reasons for exclusion of doses as OEs.

### Prevalence and type of medication administration errors

**Fig. 1:**
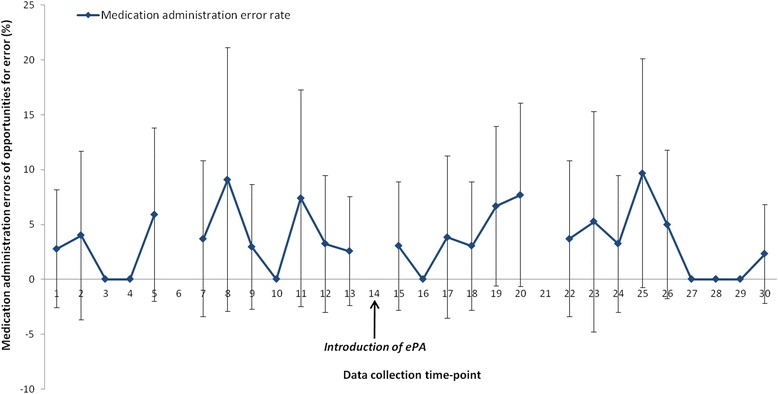
Time series graph showing observed medication administration error (MAE) rates and 95% confidence intervals pre-ePA (paper) and post-ePA (ePA only) (*ePA electronic prescribing and administration*)

Table 2 summarises MAE rates pre- and post-ePA; the researcher informed the nurse of four MAEs, one of which was pre-ePA and three were post-ePA. No significant difference in MAE prevalence was observed post-ePA. Figure 1 shows MAE rates over time; a segmented regression analysis did not detect any changes in level or trend post-ePA.

**Table 2:** Medication administration errors observed pre- and post-ePA

	Pre-ePA (paper only; 428 OEs)		Post-ePA (ePA only; 528 OEs)			Post-ePA (ePA and paper; 597 OEs)		
	n	Percentage (95% CI)	n	Percentage (95% CI)	*P* value^a^	n	Percentage (95% CI)	*P* value^b^
Total MAEs	18	4.2% (2.3–6.1)	18	3.4 (1.9–5.0)	0.64	24	4.0% (2.4–5.6)	0.99
MAEs excluding omission due to unavailability of drug	12	2.8% (1.2–4.4)	11	2.1% (0.9–3.3)	0.61	12	2.0% (0.9–3.1)	0.54
Only MAEs due to unavailability of drug	6	1.4% (0.2–2.5)	7	1.3% (0.3–2.3)	0.86	12	2.0% (0.9–3.1)	0.62

*CI* confidence interval, *OE* opportunity for error, *MAE* medication administration error, *n* number of errors, *ePA* electronic prescribing and administration system

^a^ χ^2^ test for association between pre-ePA and post-ePA (ePA only) MAE rate

^b^ χ^2^ test for association between pre-ePA and post-ePA (ePA and paper) MAE rate

**Fig. 2:**
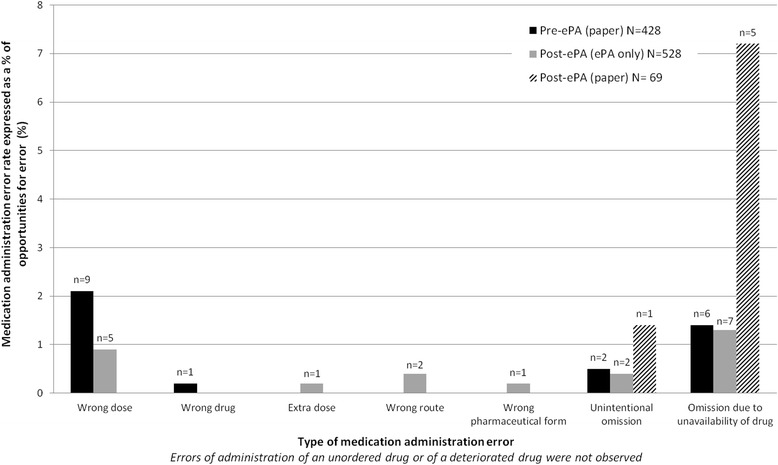
Types of medication administration errors observed associated with either paper or ePA (*ePA electronic prescribing and administration*)

MAEs observed pre-ePA comprised wrong dose, wrong drug and omission errors; it was only post-ePA that errors involving extra dose, wrong route and wrong pharmaceutical form were observed (Fig. 2). Pre-ePA, there were 12 OEs concerning oral liquid preparations, 4 (33%) of which were MAEs. Post-ePA, there were 28 liquid OEs and no related MAEs. Table 3 describes some MAEs observed; Additional file 2 presents the full list.

**Table 3:** Examples of medication administration errors observed

Paper or ePA prescribing	Error type (definitions based on existing work [30])	Drug(s) involved in error	Field notes for additional context where relevant
Pre-ePA (paper)	Wrong dose	5 mg of morphine sulphate solution administered instead of 2.5 mg	The prescribed dose was “2.5 mg”. The nurse erroneously drew 2.5 ml of 10 mg/5mlsolution instead of 1.25 ml into an oral syringe. The quantity in the syringe was checked by a second nurse and a student nurse was also observing.
Pre-ePA (paper)	Wrong dose	312.5 mg of co-amoxiclav liquid administered instead of 625 mg	The nurse originally read the prescribed dose as “625 mg”. Then they read the concentration of co-amoxiclav on the bottle (250 mg/62.5 mg in 5 ml) and concluded that the prescribed dose actually read 62.5 mg as stated on the bottle of co-amoxiclav, not 625 mg. They informed the student nurse that the dose correlates to the smaller of the two numbers stated on the co-amoxiclav bottle (62.5 mg). Therefore 5 ml was prepared, the researcher intervened.
Pre-ePA (paper)	Wrong dose	12.5 mg of spironolactone administered instead of 25 mg	The original prescribed dose was 12.5 mg which had then been amended by the prescriber by scoring through the dose and re-writing “25 mg” next to the old dose. The rewritten dose was potentially unclear and interpreted as 12.5 mg.
Post-ePA (paper)	Unintentional omission	Ramipril 2.5 mg	The nurse did not notice this drug was written on a new drug chart, the administration box was left blank. The paper chart was then transcribed to ePA and the next dose was prescribed for the following morning, so the dose was omitted.
Post-ePA (ePA)	Wrong dose	25 mg of metolozone administered instead of 2.5 mg	The nurse prepared five 5 mg tablets instead of cutting one tablet in half. The researcher intervened. The nurse stated that they read the dose specifically as they were not familiar with the drug. They could not see the decimal place on the computer screen and therefore read 25 mg as the dose.
Post-ePA (ePA)	Wrong form	Venlafaxine 75 mg modified release administered instead of immediate release	The medication administered was the patient’s own, therefore it is likely that the prescription was incorrect although the nurse did not notice this.
Post-ePA (ePA)	Wrong route	Furosemide 40 mg oral administered instead of intravenous dose	The nurse prepared oral furosemide for administration. The researcher intervened and the nurse stated that they had not noticed the route of administration.
Post-ePA (ePA)	Wrong route	Atropine 1% eye drops administered in eyes instead of sublingually	The eye drops were being used off-label and prescribed via sublingual route although administered in each eye. The researcher intervened and the nurse stated they had not noticed the additional instructions specifying the route of administration. The researcher informed the nurse after administration to the eye.

When comparing omissions due to drug unavailability pre-ePA with the small number of paper-based OEs post-ePA, there was a higher rate post-ePA. Pre-ePA there were 6 (1.4%) of 428 OEs, and post-ePA (on paper) there were 5 (7.2%) of 69 OEs (*p* = 0.01; chi-squared test).

### Prevalence and types of documentation discrepancies

**Fig. 3:**
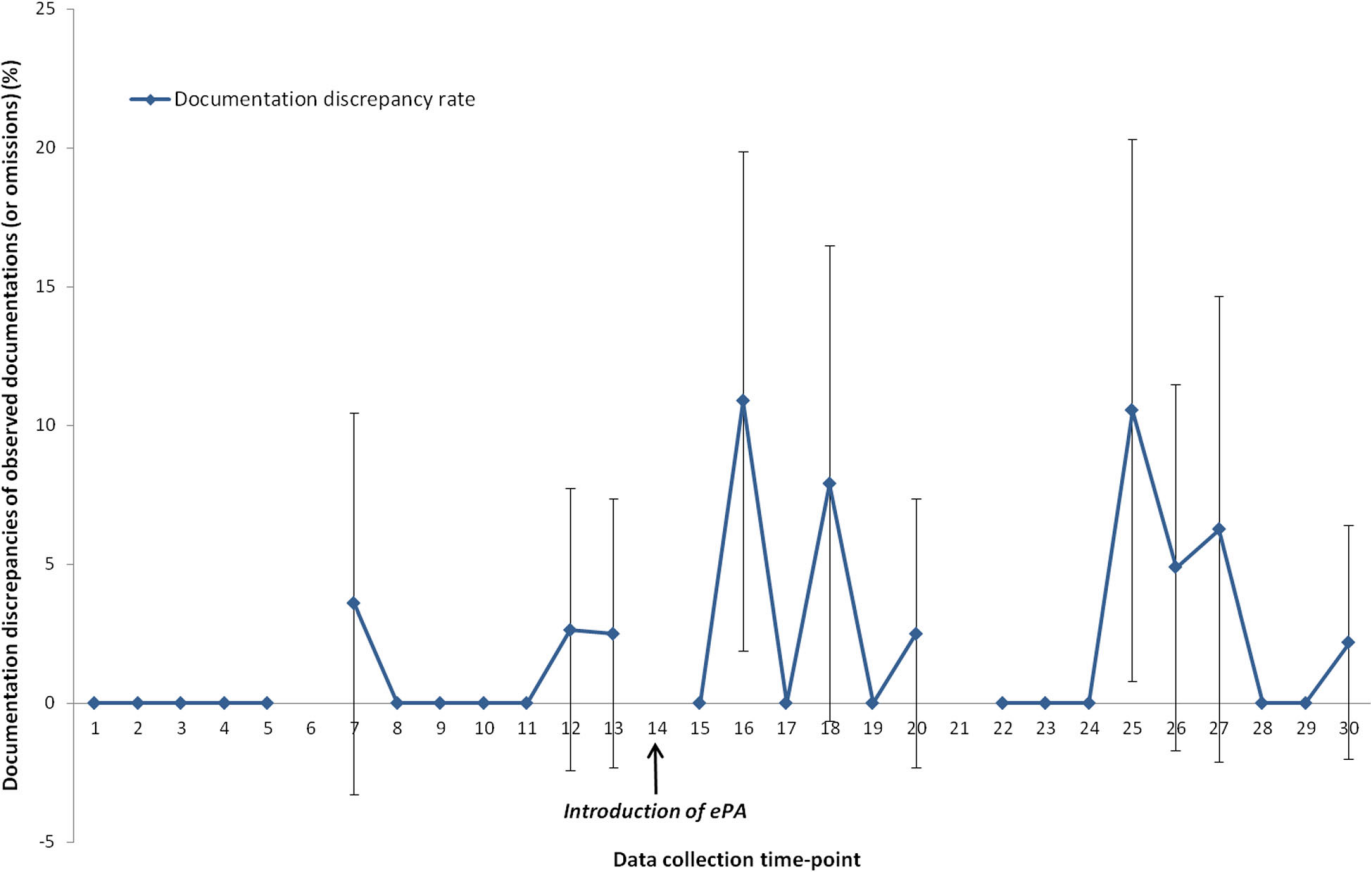
Time series graph showing observed documentation discrepancy (DD) rates and 95% confidence intervals pre-ePA (paper) and post-ePA (ePA only) (*ePA electronic prescribing and administration*)

Pre-ePA there were 460 observed documentations of administrations (or omissions), of which 5 (1.1%; CI 0.1–2.0%) were DDs. Post-ePA, using ePA only, there were 18 (3.2%; CI 1.8–4.7%) DDs of 557 observed documentations (*p* = 0.04; chi-squared test). Post-ePA, including both paper and ePA, there were 19 (3.0%) (CI 1.7–4.4%) DDs of 626 observed documentations (*p* = 0.05; chi-squared test). Figure 3 demonstrates DDs over time. Segmented regression analysis did not detect any change in level or trend.

Pre-ePA, DDs comprised dose not administered and the administration box left blank (*n* = 1), and the dose not being administered although signed to suggest it had been (*n* = 5). Post-ePA (ePA only), types of DDs comprised dose not being administered and the administration box left blank (*n* = 2), the dose being administered although the administration box was not signed (*n* = 2), and the dose not being administered although signed to suggest it had been (*n* = 14). Additionally post-ePA on paper, there was one discrepancy observed where the dose was administered but the administration box left blank.

### Changes to medication administration processes following ePA implementation

#### Working practices

Pre-ePA, it was noted that on the ward, nurses systematically marked the relevant medication administration box with a ‘dot’ to signify preparing each dose and signed for administration immediately only after the patient consumed the dose. With ePA, less consistent self-checking behaviour was observed with some nurses signing for administration following preparation rather than patient consumption. Another change observed was that pre-ePA, nurses collated any drug-charts that had outstanding jobs, such as to order or retrieve medication, and dealt with these after the medication round. With ePA, some nurses dealt with the problem there and then, others would sign for administration but make a hand-written list of outstanding jobs to address afterwards.

#### Response to warnings and alerts

With ePA, nurses were observed acting on electronic warnings and alerts that they interpreted as an indication of a prescribing error and subsequently omitted the dose concerned, which may not have been appropriate. In one instance a nurse omitted an antibiotic dose at their discretion with an intention to inform the doctors, because an alert indicated the prescription had exceeded the intended duration; this was not included as an MAE. Another occurred when an alert warned of a duplication of levothyroxine so the dose was omitted, however the intended dose alternated every other day and the duplication was therefore intentional; this was included as an MAE.

#### Infrastructure

On some occasions when using ePA, COWs were not charged and therefore had to remain stationary plugged into the electricity supply. This meant that nurses would walk back and forth to the stationary COW or were required to use two trolleys: a standalone COW plus the COW with medication storage.

## Discussion

The overall MAE rate was similar to previously reported UK MAE rates [8] with no significant difference following introduction of ePA. Findings suggest that ePA encourages certain types of MAEs and mitigates others. DDs increased with ePA, this is likely to be influenced by the adoption of new working practices.

### Medication administration errors

A key advantage of ePA is that it can potentially ensure inclusion of essential information that may be omitted on paper, and wrong dose errors caused by ambiguous handwriting are eliminated [18, 19]. MAEs associated with misinterpretation of liquid concentrations were observed using paper prescribing, despite there being more liquid OEs post-ePA. Pre-ePA, local policy stipulated that liquid formulations should be prescribed by dose not volume. However using ePA, for many liquids, concentration is specified and subsequently doses are prescribed in volume. This negates any potential volume miscalculations by nurses when preparing the dose. Additionally, MAEs occurred on paper where prescribers amended a dose on multiple occasions, leading to misinterpretation of the intended dose. When changing doses on ePA, a new medication order is generated therefore eliminating this problem. Although re-ordering on ePA in this way has been reported to cause unintentional order duplications [12, 20], this was only observed once in the present study and was noticed by the nurse so did not lead to an MAE. Nevertheless, adjusting doses caused one MAE. This occurred when a nurse purposely delayed a dose of fludrocortisone until the end of the round; on returning to the ePA chart, they could no longer see the outstanding dose, so it was omitted. This was because in the interim, it had been re-prescribed at a higher dose, but to commence the following day. Similar types of MAEs were introduced when transferring from paper to ePA, particularly omissions due to the unavailability of the drug. Nurses would temporarily omit a dose with the intention to administer it when it was supplied from pharmacy. However, if in the interim the paper chart was transcribed to ePA, the dose was often omitted until the next day. Similar MAEs have previously been reported with a hybrid of two systems [11, 13, 21]. Piloting and phased roll-out of ePA may contribute to such errors due to simultaneous use of ePA and paper systems within the organisation.

Findings suggest ePA may introduce new types of MAEs involving extra dose, wrong route and wrong pharmaceutical formulation. When these errors occurred, nurses had generally not noticed additional relevant information, such as specification of modified release, or instructions to administer eye drops by an alternative route. A reason why similar errors were not observed on paper may be because of the visual cues that a paper chart allows, such as use of coloured ink or highlighting key information. These cues are lost using ePA [11]. While many ePA systems utilise visually noticeable warnings and alerts, these are largely targeted at prescribers [22]. While ePA can potentially improve clarity and completeness of medication orders, the small on-screen computerised text can be misinterpreted by certain users, as observed when a nurse misread a dose of metolozone as 25 mg instead of the intended 2.5 mg.

#### Documentation discrepancies

DDs in which a dose had not been administered but was signed to suggest it had been were more common with ePA. These were often due to a nurse signing following preparation, and if administration was subsequently unsuccessful due to the patient declining medication or being unable to take the dose, documentation not being amended. A previous study has reported similar increases in DD with ePA [16]. In our study, nurses using paper charts demonstrated a self-checking procedure that facilitated signing only once a patient consumed a dose. The ePA documentation process does not allow for an equivalent resilience strategy. This suggests the design of electronic systems is based on stated policy rather than consideration of actual practice or the more nuanced functions afforded by paper drug-charts [23, 24]. While documentation can be amended retrospectively with ePA, it may be perceived to be time-consuming or tedious [12, 21]; it was not possible via a simple “undo” function. Nevertheless, as the system did facilitate a two-person check for narcotics, it may be possible to develop a two-stage process to document preparation and administration separately. In the present study, nurses also perceived the need to document administration on time and perhaps felt uncomfortable that exact times of administration were recorded with ePA. Indeed as ePA introduces a more robust audit trail and associated monitoring, nurses may perceive their practice to be under scrutiny, a concept previously described as “technovigilance” [25]. This may explain why nurses would sign for administration when a patient or drug was unavailable and make a note to complete administration later. This may also encourage the well-reported paper-persistence that occurs with ePA [26].

#### Strengths and limitations

The main strength is that the observational methods used are considered to be reliable and accurate to detect MAEs [27]. Additionally, data were collected throughout the week to ensure findings represented practice on both weekdays and weekends. Although it is possible that nurses changed their practice because of the presence of an observer, this has not been shown to affect MAE rates [28]. Improved reliability could have been achieved by introducing a second observer and testing for inter-rater reliability; however, this could have been perceived as threatening for the nurses, and was not feasible within the resources available. The generalisability of findings is limited because we studied one ward where a relatively narrow range of medications was used. It is likely that in other environments, different MAEs may be observed. It is also possible that findings reflect the nature of a pilot implementation phase, where there may be additional monitoring, assistance and scrutiny of the new system on pilot wards.

The main limitation was the relatively small sample size. Because of the ward setting and patient group, fewer than the recommended 100 OEs were recorded at each time-point [17]; subsequently it may not have been possible to detect any longitudinal changes or ‘settling in time’ using interrupted time-series analysis. A post-hoc power calculation based on a two group comparison between paper and ePA, using alpha of 0.05 and beta of 0.8, suggests it would have only been possible to detect a change in MAE rate from 4.2% to 1.3% or 8.7%. Furthermore, wide variability in individual nurses’ practices and adaptability to ePA was observed, as documented previously [29], and our small sample size precluded formal exploration of clustering of error rates by nurse. Time-intervals were not always equal and two data points were missed, further limiting our time-series analysis. Although it was not possible to detect changes over time, descriptive comparisons of pre- and post-ePA identified a difference in the types of MAEs, an important finding when considering ePA implementation.

#### Recommendations for practice

ePA systems should be designed to ensure essential information is easily noticed and interpreted at the point of medication administration. Similarly, ePA functionality should reflect working practices rather than stated policy, such as for the self-checking process whereby nurses differentiated between dose preparation and consumption on paper drug-charts. Additionally, amending ePA documentation retrospectively should be made more streamlined to aid nurses to accurately document administration.

Hospital organisations and healthcare professionals should be aware of the potential for errors when doses are adjusted on ePA, and omissions associated with hybrid paper and electronic systems. The period of such hybrid environments during piloting and phased roll-out should be minimised to reduce the risk of potentially dangerous errors associated with patients transferring between wards using paper and ePA.

#### Recommendations for future research

Larger studies exploring the effect of ePA on MAEs should be conducted in different ward environments where a wider range of drugs, including intravenous medications, are used; analysis of MAE severity should also be considered. Future studies should also document error rates by nurse to allow analysis to take into account any possible clustering of error rates. It would be of value to simultaneously explore prescribing errors, particularly as these can lead to administration errors. In the present study, there were three MAEs observed with ePA that were likely to have been caused by prescribing errors, although a sensitivity analysis excluding these as OEs and MAEs did not affect our conclusions. To further explore the extent to which MAEs are attributable to ePA, the causes of MAEs should be explicitly explored in parallel with future quantitative work.

## Conclusion

Findings from this small study suggest no difference in the prevalence of MAEs between paper and electronic prescribing, although there may be new types of MAEs introduced with ePA. The completeness of medication orders on ePA eliminates ambiguities around intended doses, however MAEs occurred when essential instructions to aid accurate preparation and administration of medication was not displayed clearly. A statistically significant increase in documentation discrepancies post-ePA was largely due to new working practices and nurses not amending documentation as needed. To improve medication administration safety, ePA systems should be designed to reflect actual working practices rather than stated policy. There should also be an increased awareness of the types of MAEs and DD associated with ePA and with hybrid paper and electronic systems.

## Additional files

Additional file 1: Reasons for exclusion as an opportunity for error: details of the reasons why doses were excluded as opportunities for error pre- and post-ePA. (DOCX 13 kb)

Additional file 2: Brief descriptions of medication administration errors observed: a full list of the medication administration errors observed pre- and post-ePA. (DOCX 18 kb)

## Abbreviations

COW: Computer on wheels
DD: Documentation discrepancy
ePA: Electronic prescribing and administration
MAE: Medication administration error
OE: Opportunity for error
