# Supplementary material for: The impact of a hospital electronic prescribing and medication administration system on medication administration safety: an observational study
Source: BMC Health Serv Res. 2017 Aug 9;17:547. doi: 10.1186/s12913-017-2462-2 (PMC5549345; doi:10.1186/s12913-017-2462-2)
Supplement: Supplementary file 1 — Reasons for exclusion as an opportunity for error: details of the reasons why doses were excluded as opportunities for error pre- and post-ePA. (DOCX 13 kb) [file 12913_2017_2462_MOESM1_ESM.docx]

**Reasons for exclusion as an opportunity for error**

| **Reason dose was excluded as opportunity for error** | **Pre-ePA (n)** | **Post-ePA (n)** |
| --- | --- | --- |
| Patient declined before dose preparation | 12 | 16 |
| Omission due to nurse’s clinical judgement | 16 | 3 |
| Patient prepared and self-administered the dose | 4 | 0 |
| No space to document on paper drug-chart until re-written | 1 | 0 |
| Researcher was unable to observe in enough detail | 20 | 27 |
| **Total** | **53** | **46** |

*ePA electronic prescribing and administration*
