# Supplementary material for: The impact of a hospital electronic prescribing and medication administration system on medication administration safety: an observational study
Source: BMC Health Serv Res. 2017 Aug 9;17:547. doi: 10.1186/s12913-017-2462-2 (PMC5549345; doi:10.1186/s12913-017-2462-2)
Supplement: Supplementary file 2 — Brief descriptions of medication administration errors observed: a full list of the medication administration errors observed pre- and post-ePA. (DOCX 18 kb) [file 12913_2017_2462_MOESM2_ESM.docx]

**Brief descriptions of medication administration errors observed**

| **Medication administration errors associated with paper prescribing (pre-intervention unless specified*)** | | |
| --- | --- | --- |
| **Error type (definitions based on existing work [16])** | **Description of error** | **Field notes for additional context where relevant** |
| **Wrong dose** | 5mg of morphine sulphate solution administered instead of 2.5mg | The prescribed dose was “2.5mg”. The nurse erroneously drew 2.5ml of 10mg/5mlsolution instead of 1.25ml into an oral syringe. The quantity in the syringe was checked by a second nurse and a student nurse was also observing. |
|  | 312.5mg of co-amoxiclav liquid administered instead of 625mg | The nurse originally read the prescribed dose as “625mg”. Then they read the concentration of co-amoxiclav on the bottle (250mg/62.5mg in 5ml) and concluded that the prescribed dose actually read 62.5mg as stated on the bottle of co-amoxiclav, not 625mg. They informed the student nurse that the dose correlates to the smaller of the two numbers stated on the co-amoxiclav bottle (62.5mg). Therefore 5ml was prepared, the researcher intervened. |
|  | 12.5mg of spironolactone administered instead of 25mg | The original prescribed dose was 12.5mg which had then been amended by the prescriber by scoring through the dose and re-writing “25mg” next to the old dose. The rewritten dose was potentially unclear and interpreted as 12.5mg. |
|  | 4mg of perindopril administered instead of 8mg | The nurse was particularly busy as there was only one nurse on the ward due to staff shortage. Subsequently there were many distractions and interruptions during the drug round. One 4mg tablet was administered instead of two tablets. |
|  | 80mg of gliclazide administered instead of 40mg | The 80mg tablet should have been halved |
|  | 40mg of furosemide administered instead of 20mg | The 40mg tablet should have been halved. The nurse expressed that they were rushed to complete the drug round after spending a long time with one patient |
|  | 60mg of isosorbide mononitrate MR administered instead of 90mg | One and a half tablets should have been administered |
|  | 1 sachet of Movicol administered instead of 2 | - |
|  | 1 tablet of vitamin B compound strong administered instead of 2 | **-** |
| **Wrong drug** | 10ml senna liquid administered instead of lactulose | Nurse went to retrieve lactulose from the treatment room without the drug chart and returned with senna liquid instead. Nurse was accompanied by student nurse. |
| **Unintentional omissions** | Ferrous fumarate 210mg | Student nurse was preparing drugs in medication pot, the nurse supervising her checked and signed but did not notice ferrous fumarate had not been administered. |
|  | Nystatin 1ml | Medication administration was omitted in error and nurse signed to say it was administered |
|  | *Ramipril 2.5mg (post-intervention) | Nurse did not notice this drug was written on a new drug chart, the administration box was left blank. The paper chart was then transcribed to ePA and the next dose was prescribed for the following morning, so the dose was omitted. |

| **Medication administration errors associated with electronic system** | | |
| --- | --- | --- |
| **Error type** | **Description of error** | **Field notes for additional context where relevant** |
| **Wrong dose errors** | 375mg carbocisteine administered instead of 750mg | One capsule was administered instead of two. Nurse was using a computer on wheels with integrated medication drawers. |
|  | Adcal D3® tablet administered instead of caplet | One tablet contains calcium carbonate 1500mg and vitamin D3 400 international units and one caplet is half the strength. The nurse did not notice the strength or formulation prescribed on the prescription. It is likely that this was in fact a prescribing error and tablets were intended to be prescribed because it was newly started in the hospital, the standard formulation is tablets and two caplets would usually be the correct dose. |
|  | 60mg of isosorbide mononitrate administered instead of 30mg | One 60mg tablet should have been halved |
|  | 60mg of isosorbide mononitrate administered instead of 30mg | One 60mg tablet should have been halved |
|  | 25mg of metolozone administered instead of 2.5mg | The nurse prepared five 5mg tablets instead of cutting one tablet in half. The researcher intervened. The nurse stated that they read the dose specifically as they were not familiar with the drug. They could not see the decimal place on the computer screen and therefore read 25mg as the dose. |
| **Extra dose** | Chloramphenicol 0.5% eye drops administered in both eyes instead of right eye only | The nurse was using the electronic system for the first time and was being supervised by a pharmacy support staff to help with using the system. |
| **Wrong form** | Venlafaxine 75mg modified release administered instead of immediate release | The medication administered was the patient’s own, therefore it is likely that the prescription was incorrect although the nurse did not notice this. |
| **Wrong route** | Atropine 1% eye drops administered in eyes instead of sublingually | The eye drops were being used off-label and prescribed via sublingual route although administered in each eye. The researcher intervened and the nurse stated they had not noticed the additional instructions specifying the route of administration. The researcher informed the nurse after administration to the eye. |
|  | Furosemide 40mg oral administered instead of intravenous dose | Nurse prepared oral furosemide for administration. The researcher intervened and the nurse stated that they had not noticed the route of administration. The researcher intervened. |
| **Unintentional omissions** | Calcichew D3 1 tablet | Nurse did not notice calcichew D3 was not administered, the patient was on a large number of medications |
|  | Omeprazole 20mg | Nurse did not notice omeprazole had not been administered although signed to indicate it was administered |

| **Omissions due to unavailability of drug** | | |
| --- | --- | --- |
| **Pre-intervention (paper)** | **Post-intervention (paper)** | **Post-intervention (electronic system)** |
| Baclofen liquid 5mg Doxazosin 1mg Bendroflumethiazide 2.5mg Ferrous sulphate 200mg Tiotropium 18micrograms Elipta Relvar inhaler 1 puff | Nicorandil 10mg Linagliptin 5mg Paroxetine 30mg Carbocisteine 375mg Ranitidine 150mg | Hypromellose 0.3% eye drops Formoterol 12micrograms inhaler 1 puff Tiotropium inhaler 18 micrograms Symbicort 400/12 1 puff Indapamide 2.5mg Fludrocortisone 100micrograms -  Lansoprazole 15mg |
